# Clinical Characteristics and Risk Factors for Allergic Rhinitis in Children with Epistaxis

**DOI:** 10.1155/2023/6731414

**Published:** 2023-08-31

**Authors:** Jing Qing, Yili Cai, Shixiong Tang, Yaowen Wang

**Affiliations:** ^1^Department of Otorhinolaryngology, Ningbo First Hospital, Ningbo 315000, Zhejiang, China; ^2^Department of Acupuncture, Ningbo First Hospital, Ningbo 315000, Zhejiang, China

## Abstract

**Background:**

Epistaxis is frequently observed in children with allergic rhinitis. However, few studies have addressed the clinical characteristics and risk factors for allergic rhinitis in children with epistaxis. This study aimed to describe the factors associated with allergic rhinitis in children with epistaxis.

**Methods:**

In total, we recruited 80 children (aged 3–14 years) who presented with epistaxis at a tertiary hospital between January 2014 and January 2022. The follow-up duration was at least 3 months, and we performed a multivariate logistic regression analysis to identify the risk factors for allergic rhinitis.

**Results:**

Among the 80 children examined, 57 (71.25%) had allergic rhinitis. Epistaxis mainly occurred in autumn in children with allergic rhinitis; in contrast, it mostly occurred in summer in children without it (*P* = 0.029). Mites are common allergens for allergic rhinitis in children with epistaxis; the univariate analysis revealed significant differences between allergic-rhinitis group and nonallergic-rhinitis group in the number of allergens (*P*  <  0.001) and total IgE (*P*  <  0.001). The difference in severity of nasal symptoms between the two groups was statistically significant and included nasal obstruction (*P*  <  0.001), rhinorrhea (*P*  <  0.001), sneezing (*P*  <  0.001), and nasal itching (*P*  <  0.001). After adjusting for potential confounders, the severity of rhinorrhea symptoms was found to be associated with an increased risk of allergic rhinitis in children with epistaxis (odds ratio: 3.86; 95% confidence interval: 1.61–9.26; *P* = 0.003).

**Conclusions:**

Observing the onset season, number of allergens, total IgE, and nasal symptoms in cases of epistaxis could suggest the presence of associated allergic rhinitis and reduce the number of missed diagnoses; antiallergic drugs could help control epistaxis in these cases.

## 1. Introduction

Epistaxis is a common complaint encountered in children in the otorhinolaryngology department, and the incidence of epistaxis is the highest in patients aged between <10 and >70 years [1, 2]. Nearly half of the children present at least one episode of epistaxis, and more than 60% of the general population report at least one episode during their lifetime [3, 4]. These events affect approximately 64% of children aged 11–15 years, 56% of those aged 6–10 years, and 30% of children younger than 5 years [4, 5]. Epistaxis in children is often spontaneous and self-limited; however, repeated attacks can lead to anxiety among children and parents. Its pathogenesis is unknown; however, infections, inflammation, injuries, air pollutants, and meteorological factors are considered the most common causes [6, 7].

Allergic rhinitis is a chronic IgE-mediated inflammation of the nasal mucosa, with a prevalence of 15.79% among Chinese children [8]. The typical presentations include nasal symptoms (rhinorrhea, congestion, itching, and sneezing), ocular symptoms (itchy and watery eyes), and general manifestations (cough and headache). The diagnosis of this disease depends on the symptoms and signs, skin-prick test, or sIgE tests. Allergic rhinitis is significantly associated with the risk of conjunctivitis, atopic dermatitis, and allergic asthma [9, 10], and it impairs the children's quality of life, concentration, productivity, and sleep [9, 11]; thus, early diagnosis and treatment of this disease are important.

Children with epistaxis related to allergic rhinitis are often seen in hospitals as they attract their parents' attention more frequently than those with other typical nasal symptoms of allergic rhinitis. However, few studies have investigated allergic rhinitis in children with epistaxis. Therefore, we performed a retrospective study to summarize the clinical characteristics and risk factors associated with allergic rhinitis in children with epistaxis.

## 2. Materials and Methods

### 2.1. Subjects

This retrospective study was conducted in the Department of Otorhinolaryngology in the Premier Hospital in Ningbo City, Zhejiang Province, China, between January 2014 and January 2022. Children aged between 3 and 14 years who presented with epistaxis were included in the study. The same associate chief physician treated all otorhinolaryngology patients, and children with a specific intranasal pathology (other than prominent vessels on the nasal septum, crusting, or irritation) were excluded from the analysis. Patients with nasal infections, tumors, cardiovascular diseases, or blood system diseases were also excluded. This study was conducted following the tenets of the Declaration of Helsinki and was approved by the Ethics Committee of the Premier Hospital in Ningbo City. Informed consent was obtained from the guardians of all study participants. The diagnosis of allergic rhinitis was based on the Guidelines for the Diagnosis and Treatment of Allergic Rhinitis [12] and Chinese Society of Allergy Guidelines for the Diagnosis and Treatment of Allergic Rhinitis [13], including (1) patients presented with watery nasal discharge, nasal obstruction, sneezing, or itching in the nose; the history and physical examination were consistent with an allergic cause; (2) positive for specific IgE to antigens, such as house dust mites; (3) the allergic status was assessed using serum-specific IgE to common inhalant and food allergens, including dust mites, pets, molds, cockroaches, eggs, milk, and beef and so on, and the IgE level above 0.35 kU/L is usually testified as a positive result.

### 2.2. Study Procedures

The clinical characteristics and risk factors collected for the analysis included the seasonal onset of disease; sex; age; test results for the number of allergens, total IgE; red blood cells, hemoglobin, leukocytes, and blood platelets; nasal obstruction; rhinorrhea; sneezing; nasal itching; need for medical attention; anemia; epistaxis severity score (ESS); and family history. According to the allergic rhinitis and its impact on asthma guidelines and Chinese Society of Allergy Guidelines for Diagnosis and Treatment of Allergic Rhinitis, the severity of allergic rhinitis symptoms is classified as intermittent mild, intermittent moderate-severe, persistent mild, or persistent moderate-severe [12, 13]. Intermittent and persistent symptoms are defined as symptoms lasting less or more than 4 days/week or 4 weeks/year, respectively. Symptoms with no impact on daily life and sleep are considered mild, whereas those affecting them are defined as moderate to severe.

A visual analog scale (VAS) of individual nasal symptoms ranging from 0 (minimal) to 10 (extremely bothersome) was used to assess their severity. Individuals with scores of 0–4 were considered to have mild symptoms, while scores of 5–10 indicated moderate/severe symptoms [14]. Epistaxis severity was assessed using the ESS, and the scoring system ranged from 0 (no epistaxis) to 10 (most severe epistaxis). Scores of 1–3 indicated mild epistaxis, 4–7 indicated moderate, and 8–10 indicated severe [14].

All patients were administered with nasal isotonic saline irrigation and aureomycin ointment to maintain the nasal cavity's cleanliness and moisture. After the blood test results were obtained, the patients diagnosed with allergic rhinitis were instructed to avoid allergens. These patients were also administered the oral antihistamine cetirizine (0.5 ml per day after dinner for 2 weeks in children 2–6 years of age and 1 ml per day after dinner for 2 weeks in children >6 years) and mometasone (adults and children>12 years of age: 2 sprays (100 *μ*g) per nostril q.d; children of 3–11 years of age: 1 spray (50 lg) per nostril q.d, and it was administered in the morning). Each parent or guardian was trained on the proper use of nasal saline irrigation, aureomycin ointment, and nasal steroid spray to avoid drug-induced epistaxis. Patients and guardians were told not to plug their nostrils with tissue to stop bleeding, gently wash their face, and hold the nose for 3-5 minutes when bleeding. Sublingual desensitization with *Dermatophagoides farinae* drops was provided to patients who were allergic only to mites, older than 5 years, and willing to continue the desensitization for more than 3 years. All patients were followed up for 3 months, except those treated with sublingual desensitization, who needed long-term follow-up. VAS, ESS, and nasal endoscopy were performed on the first visit and 3 months after treatment.

### 2.3. Statistical Analysis

All statistical analyses were performed using IBM SPSS Statistics version 26 software (IBM Corp, Armonk, NY, USA). Continuous data that followed normal or nonnormal distribution were described using the mean ± standard deviation or median (quartiles), respectively. The differences between the groups were assessed using the Student's *t*-test or Mann–Whitney *U* test. The categorical data were described using event and frequency, and the differences between groups were examined using the chi-square or Cochran–Mantel–Haenszel tests. The Wilcoxon signed-rank test was used to compare the VAS scores of each nasal symptom before and after treatment. A multivariate logistic regression analysis was used to identify the potential risk factors for allergic rhinitis accompanying epistaxis. The tests were two-sided, and the values of *P*  <  0.05 were considered statistically significant.

## 3. Results

In total, 80 patients were included in the analysis. The age range was 3–14 years, and the median age was 9.0 years; 52 (65.0%) patients were males, and 28.75% had a family history. Fifty-seven participants (71.25%) met the diagnostic criteria for allergic-rhinitis and comprised the corresponding group, whereas 23 (28.75%) were included in the nonallergic-rhinitis group. 8 patients experienced symptoms and signs consistent with allergic rhinitis, though the serum sIgE test results were negative. The baseline characteristics of the patients examined are shown in Table 1. There were no significant differences between the two groups in terms of sex (*P* = 0.623), age (*P* = 0.348), red blood cell count (*P* = 0.094), hemoglobin level (*P* = 0.678), leukocyte count (*P* = 0.291), and blood platelet count (*P* = 0.882). Moreover, the incidence of the need for medical attention (*P*  >  0.99), anemia (*P* = 0.946), and family history (*P* = 0.738) between the study groups was not significantly different. In contrast, there was a significant difference between the study groups in the number of allergens (*P*  <  0.001) and total IgE (*P*  <  0.001).

**Figure 1:**
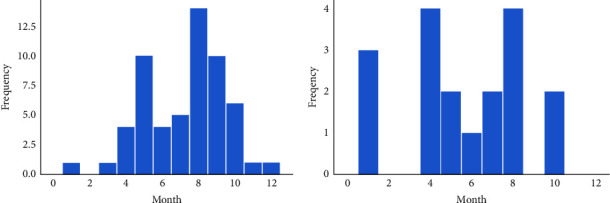
Histograms of the number of children with epistaxis in the allergic (a) and nonallergic (b) groups during different months.

We noted that the incidence of epistaxis in the allergic-rhinitis group was at its highest in autumn (50.88%), whereas in the nonallergic-rhinitis group it was at its highest in summer (30.43%).  Figure 1 shows a histogram of the number of patients with epistaxis in the different months. In the allergic-rhinitis group (Figure 1(a)), epistaxis occurred mainly in August (14 cases), followed by September and May (both 10 cases), and most patients were allergic to mites. In the nonallergic-rhinitis group (Figure 1(b)), the highest incidence of epistaxis was observed in August and April (both 4 cases), followed by January (3 cases). We noted a significant difference between the two groups in the season of onset (*P* = 0.029).

In addition, previous medical records showed that 10 patients visited our hospital twice and one patient three times for recurrent epistaxis, and they were not diagnosed with allergic rhinitis at the first or second visits. The interval from their first visit to the final diagnosis ranged from 11 days to 5 years, with a mean of 2.2 years. Before the diagnosis of allergic rhinitis, all patients were treated with aureomycin ointment and nasal saline irrigation; however, epistaxis still occurred intermittently. For patients in the allergic-rhinitis group, the most common inhalant allergens were *Dermatophagoides farina* (*D. farina*) and *Dermatophagoides pteronyssinus* (*D. pteronyssinus*), having positive results in 46 patients (80.7%), and the most common food allergens were milk (28.1%), egg white (26.3%), and beef (21.1%). 27 patients (47.4%) were sensitive to one allergen, 13 (22.8%) to two allergens, 12 (21.0%) to three, 4 (7.0%) to four, and 1 (1.8%) was sensitive to five allergens. The VAS of four nasal symptoms before treatment was summarized in Table 1. All patients with allergic rhinitis presented with a persistent mild form of the disease. Moreover, we noted a significant difference between allergic-rhinitis group and nonallergic-rhinitis group in the severity of nasal obstruction (*P*  <  0.001), rhinorrhea (*P*  <  0.001), sneezing (*P*  <  0.001), and nasal itching (*P*  <  0.001).

The severity of epistaxis in both groups was mainly mild. Four patients in the allergic-rhinitis group and one in the other group exhibited moderate symptoms, and no severe cases were noted. None of the patients in either group described their epistaxis intensity as gushing or pouring; mostly it was dripping or outflowing. None of the patients received red blood cell transfusions. Blood tests showed that seven patients (12.28%) in the allergic-rhinitis group and two (8.70%) in the nonallergic-rhinitis group had mild anemia. The platelet count and coagulation function were normal. There were no significant differences between the groups in terms of the frequency (*P* = 0.774), duration (*P* = 0.664) of epistaxis, and ESS score (*P* = 0.528).

**Figure 2:**
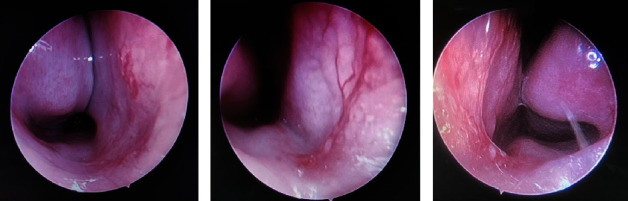
Nasal endoscopic manifestations of epistaxis in a patient with allergic rhinitis.

**Figure 3:**
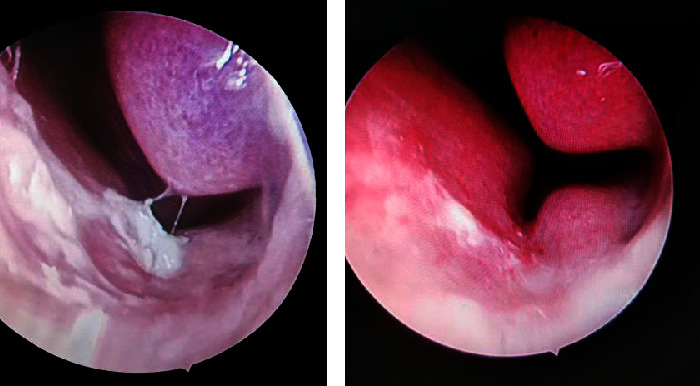
Nasal endoscopic manifestations of epistaxis in a nonallergic patient.

Allergic shiners (13 cases) and facies (11 cases) were observed in the allergic-rhinitis group. Endoscopy revealed hypervascularity of the nasal septal mucosa with vascular dilatation and tortuosity. Additional symptoms included mucus on the middle turbinate, possibly accompanied by pale or slightly cyanotic swollen mucosa and hypertrophic inferior turbinate (Figure 2). A cobblestone appearance in the posterior pharyngeal wall was common.  Figure 3 shows prominent vessels with crusts in the anterior septal region of a nonallergic patient. Mucous secretion and adenoid hypertrophy were occasionally present in patients from both groups.

Furthermore, all patients were treated with nasal isotonic saline irrigation throughout the follow-up period, along with aureomycin ointment for 2 weeks. In the allergic-rhinitis group, oral cetirizine was administered for at least 2 weeks and then discontinued, whereas the nasal steroid spray was used for 2 months and then stopped within 1 month after progressively reducing the dose. In addition, three patients underwent sublingual desensitization. Finally, after adjusting for potential confounders, we noted that the probability of allergic rhinitis diagnosis was higher in subjects with more severe rhinorrhea (odds ratio: 3.86; 95% confidence interval (CI): 1.61–9.26; *P* = 0.003).

## 4. Discussion

The present study aimed to describe the clinical characteristics of patients with epistaxis related to allergic rhinitis. Eighty children were selected using a broad range of characteristics. The age and sex of the patients in our study were similar to those of the population of previous studies that investigated the clinical characteristics, treatment, and prognosis of epistaxis [1, 15–21]. Moreover, most patients included had been diagnosed with allergic rhinitis, suggesting that patients with allergic rhinitis had a higher incidence of epistaxis than those without this allergy [22]. We observed significant differences between allergic-rhinitis group and nonallergic-rhinitis group regarding the disease onset season, total IgE, severity of nasal obstruction, rhinorrhea, sneezing, and nasal itching. After adjusting for potential confounders, we noted that the epistaxis in the allergic-rhinitis group presented with more severe rhinorrhea symptoms.

Several studies have addressed the potential association between epistaxis and seasonality. Numerous studies have shown that the incidence of epistaxis is related to seasonal variations, mostly occurring in winter [23–26]. Shay et al. observed that pediatric epistaxis occurred mainly during the spring and summer months, in contrast with previous reports in the literature, and the most severe episodes of epistaxis occurred during winter and spring [18]. Lu et al. observed that nosebleeding in children in Beijing mainly occurred from May to June and August to September, especially in 2017 [27]. Bray et al. reported that ambient temperature and season had no relationship with the epistaxis presentation rate [28]. Our results suggest that pediatric epistaxis related to allergic rhinitis is more likely to occur in late summer and early autumn, followed by spring; most patients with rhinitis were allergic to mites, while four patients were allergic to food or plants.

Moreover, 11 cases of recurrent epistaxis related to allergic rhinitis occurred at the same time of the year or during the season of allergic rhinitis; therefore, we speculate that epistaxis associated with allergic rhinitis has a seasonality and is related to the type of allergens. Lu et al. observed that pediatric epistaxis in Beijing mainly occurred in the months corresponding to the pollen season, suggesting a specific relationship with the outbreak of allergic rhinitis [27]. A cross-sectional survey performed by Li et al. on 6,304 patients with asthma, rhinitis, or both in 17 cities from four regions of China showed that the most common aeroallergens in this country were house dust mites, and 87.2% of patients were sensitive to one or more species of mites. In particular, the coastal regions were more susceptible to the growth of dust mites due to their high humidity and temperature [29]. Arlian et al. reported that dust mites had the highest proliferation at a relative humidity of 75%, and the number of dust mites in the northern USA varied with seasonal humidity, which is high in wet summers and low in dry winters [30, 31]. Lintner and Brame also observed that dust mite allergens are associated with seasonal variations [32]. Ningbo, a city close to the East China Sea, is humid and hot in late summer and early autumn and is favorable for mites' survival. All patients in this study lived in Ningbo, and most patients in the allergic-rhinitis group were allergic to mites; hence, this factor may be the main cause of epistaxis observed in patients with allergic rhinitis during our study's period.

Children with epistaxis did not always exhibit clearly the symptoms of allergic rhinitis, and their parents or guardians failed to notice these symptoms. We observed that nasal itching was the most evident symptom; in contrast, nasal congestion was the most neglected, possibly because the parents more easily noticed allergic salute and nasal rubbing caused by itching. In addition, the feeling of itching could be expressed more clearly by children. It was difficult for children to report nasal obstruction voluntarily, unless the stuffiness was severe. The parents often ignored this symptom because they felt that their children did not snore or open their mouths sufficiently to breathe during sleep.

Moreover, we noted that the severity of rhinorrhea symptoms was significantly related to allergic rhinitis, as already demonstrated in previous studies [33–35]. We also noted that pediatric epistaxis occurred once or more times per week, and the attack time did not exceed 5 min. Most cases of epistaxis resolve without the need for a hospital visit; however, chronic and recurrent epistaxis markedly impacts the patients' health. In our study, seven patients in the allergic-rhinitis group and two in the nonallergic group had mild anemia.

Nasal endoscopy revealed nasal vasodilatation, angiogenesis, and increased vascular permeability of the anterior nasal septum in the allergic-rhinitis group. However, the specific mechanisms linking allergic rhinitis and such changes in the nasal mucosa, leading to epistaxis, remain to be determined. Girsh thought that epistaxis was more likely to occur in children with allergic rhinitis because nasal itching could cause allergic salutes, and repeated rubbing could damage the nasal mucosa, making inflamed and friable by the vascular congestion and inflammation associated with allergic rhinitis [22]. Moreover, airway remodeling may cause changes in the nasal mucosa and blood vessels. Previous studies have revealed that airway remodeling is less extensive in allergic rhinitis than in asthma [36–40]; it involves smooth muscle hypertrophy, goblet cell hyperplasia, infiltration of inflammatory cells, and vascular remodeling.

In contrast, the mechanisms of nasal remodeling have not been established yet. As part of airway remodeling, it plays a vital role in epistaxis related to allergic rhinitis. When pathological changes occur in the blood vessels of the nasal septal mucosa, brittleness increases, and even minor injuries, such as rubbing, scratching, and sneezing, can cause blood vessel damage and epistaxis.

Most pediatric epistaxis cases are venous and tend to recur, especially if the cause is unknown. Common therapies include nasal saline irrigation and local use of ointments to moisten the nasal mucosa and reduce inflammation and crusting [41, 42]. Alternatively, silver nitrate cautery is more suitable for older children who do not respond to simple medical treatment and have prominent hemorrhagic spots [43]. Our study observed that 11 patients with a missed diagnosis of allergic rhinitis had a history of recurrent attacks, even when treated with saline and aureomycin ointment. We speculate that these methods alone have limited effects when epistaxis is related to allergic rhinitis.

Some limitations of this study should be acknowledged. First, the number of patients was small; thus, the results may not be reliable, as shown by the broad 95% CI. Second, the difference in severity of the nasal symptoms before and after treatment was examined in the allergic-rhinitis group only, and the differences between the groups were not investigated. Third, the baseline characteristics of the children examined had a wide variability, possibly affecting the treatment and prognosis of epistaxis.

## 5. Conclusions

Pediatric epistaxis related to allergic rhinitis is common, and allergic rhinitis is easily overlooked when the allergic symptoms are atypical. Therefore, otorhinolaryngologists need to collect a detailed history, including the frequency of epistaxis, season of most common attacks, and habits of allergic salute or nasal rubbing, and perform a nasal endoscopy to observe the site and characteristics of epistaxis and possible changes in the nasal mucosa, such as edema or color changes in the turbinates. If necessary, a skin-prick or serum-specific IgE test can help reduce the rate of missed diagnoses. Moreover, the severity of rhinorrhea symptoms is significantly related to allergic rhinitis in children with epistaxis. Large-scale prospective studies should be conducted to confirm these findings and develop a predictive model for epistaxis related to allergic rhinitis.

## Figures and Tables

**Table 1:** Baseline characteristics of the patients.

Variable	Overall (*N* = 80)	Group		
		Allergic rhinitis group (*N* = 57)	Nonallergic rhinitis (*N* = 23)	*P* value
Onset season				0.029
Spring	7 (8.75)	2 (3.51)	5 (21.74)	
Summer	25 (31.25)	18 (31.58)	7 (30.43)	
Autumn	35 (43.75)	29 (50.88)	6 (26.09)	
Winter	13 (16.25)	8 (14.04)	5 (21.74)	
Gender				0.623
Girl	28 (35.00)	19 (33.33)	9 (39.13)	
Boy	52 (65.00)	38 (66.67)	14 (60.87)	
Age (years)	9.00 (6.00, 12.00)	9.00 (6.00, 11.00)	10.00 (7.00, 12.00)	0.348
Number of allergen				<0.001
0	21 (26.25)	0 (0.00)	21 (91.30)	
1	29 (36.25)	27 (47.37)	2 (8.70)	
2	13 (16.25)	13 (22.81)	0 (0.00)	
3	12 (15.00)	12 (21.05)	0 (0.00)	
4	4 (5.00)	4 (7.02)	0 (0.00)	
5	1 (1.25)	1 (1.75)	0 (0.00)	
Total IgE	55.80 (22.42, 189.71)	79.61 (35.93, 292.13)	33.12 (14.80, 50.50)	<0.001
Red blood cell	4.65 (0.41)	4.70 (0.43)	4.53 (0.36)	0.094
Hemoglobin	13.00 (12.30, 14.00)	12.90 (12.30, 14.10)	13.00 (12.50, 13.60)	0.678
Leukocyte	6.96 (1.64)	6.84 (1.65)	7.27 (1.62)	0.291
Blood platelet	268.50 (236.00, 301.50)	265.00 (239.00, 303.00)	270.00 (232.00, 299.00)	0.882
Nasal obstruction severity	0.00 (0.00, 3.00)	1.00 (0.00, 5.00)	0.00 (0.00, 0.00)	<0.001
Rhinorrhea severity	1.00 (0.00, 4.00)	2.00 (1.00, 5.00)	0.00 (0.00, 0.00)	<0.001
Sneezing severity	1.00 (0.00, 4.00)	2.00 (0.00, 5.00)	0.00 (0.00, 1.00)	<0.001
Nasal itching severity	3.00 (2.00, 5.00)	3.00 (2.00, 5.00)	2.00 (0.00, 3.00)	<0.001
Frequency of epistaxis				0.774
Once per month	14 (17.50)	9 (15.79)	5 (21.74)	
Once per week	37 (46.25)	26 (45.61)	11 (47.83)	
Several per week	21 (26.25)	17 (29.82)	4 (17.39)	
Once per day	6 (7.50)	4 (7.02)	2 (8.70)	
Several each day	2 (2.50)	1 (1.75)	1 (4.35)	
Duration of epistaxis				0.664
<1 minute	51 (63.75)	36 (63.16)	15 (65.22)	
1–5 minutes	27 (33.75)	19 (33.33)	8 (34.78)	
6–15 minutes	2 (2.50)	2 (3.51)	0 (0.00)	
Need for medical attention				1.000
No	76 (95.00)	54 (94.74)	22 (95.65)	
Yes	4 (5.00)	3 (5.26)	1 (4.35)	
Anemia				0.946
No	71 (88.75)	50 (87.72)	21 (91.30)	
Yes	9 (11.25)	7 (12.28)	2 (8.70)	
ESS score	1.40 (1.00, 2.50)	1.40 (1.00, 2.50)	1.00 (1.00, 2.50)	0.528
Family history				0.738
No	57 (71.25)	40 (70.18)	17 (73.91)	
Yes	23 (28.75)	17 (29.82)	6 (26.09)	

## Data Availability

The datasets generated and/or analyzed during the current study are available from the corresponding author upon reasonable request.
